# Laparoscopic cholecystectomy for symptomatic cholelithiasis in children and adolescents: analysis of 50 cases from a single institution

**DOI:** 10.1590/acb394124

**Published:** 2024-07-22

**Authors:** Renato Queiroga de Almeida, Vicente Antonio Gerardi, José Luiz Ferreira Dias, Manuela Corrêa de Toledo Peres, Jaques Waisberg

**Affiliations:** 1 Hospital Estadual Mário Covas – Unit of Pediatric Surgery – Santo André (SP), Brazil.; 2 Faculdade de Medicina do ABC – Department of Maternal and Child Health – Santo André (SP), Brazil.; 3 Faculdade de Medicina do ABC – Department of Surgery – Santo André (SP), Brazil.

**Keywords:** Cholecystolithiasis, Cholecystectomy, Laparoscopic, Cholecystectomy, Child, Pediatric Obesity

## Abstract

**Purpose:**

To investigate the clinical characteristics of symptomatic cholecystolithiasis and laparoscopic cholecystectomy complications in pediatric patients.

**Methods:**

The medical records of 50 children and adolescents who underwent laparoscopic cholecystectomy were analyzed. We evaluated gender, age, body mass index, preoperative clinical aspects, perioperative complications, and gallstone composition.

**Results:**

Among the patients, 33 (66%) were female, and 17 (34%) were male. The mean age was 11.4 ± 3.6. All patients were diagnosed with cholecystolithiasis by abdominal ultrasonography. Twelve patients (24%) had hematological disease: eight (16%) with sickle cell anemia and four (8%) with hereditary spherocytosis. Thirteen patients (26%) were obese. Twelve patients (24%) had complicated biliary disease. During the intraoperative period, three patients (6%) had excessive bleeding in the hepatic hilum, and one had an accidental injury to the common bile duct. Three (6%) postoperative complications (acute pancreatitis, common bile duct stenosis, and intestinal obstruction) were observed. Among 28 patients (56%), 25 (50%) had cholesterol gallstones, and three (6%) had bile pigment gallstones.

**Conclusions:**

The evolution of cholecystolithiasis in the pediatric population can present serious complications, emphasizing the need to avoid temporizing cholecystolithiasis in children and adolescents because laparoscopic cholecystectomy in this group is safe, with low complication rates.

## Introduction

Cholecystolithiasis is a rare pediatric disease with a prevalence between 0.13 and 1.9%^1^. About 80% of pediatric patients with gallstones in the gallbladder remain asymptomatic during their lifetimes^2^^–^ 4.

The increase in the incidence of cholecystolithiasis in the pediatric population was registered in studies carried out in the last three decades^5^^–^ 7. The increased awareness of gallbladder disease as a cause of abdominal pain in children may be associated with the growing use of abdominal ultrasonography, consequently improving the diagnosis of cholecystolithiasis^3^^–^ 5. However, in clinical practice, the diagnosis and treatment of this condition in children and adolescents are based on studies of small populations^3^^,^ 4.

Although hemolytic diseases (sickle cell anemia, hereditary spherocytosis, and thalassemia) have been identified as risk factors, the prevalence of pediatric cholecystolithiasis has increased proportionally with the recent childhood obesity epidemic^8^^,^ 9. The most likely cause for this increase in cholecystolithiasis in children and adolescents would be the rise in this population’s mean body mass index (BMI)^6^^,^ 10 ^,^ 11, with a third of children being overweight or obese, which is considered the cause of most cholecystectomies performed in pediatric patients^12^. Other reasons for cholecystolithiasis have included a cholesterol-rich diet, cephalosporins, total parenteral nutrition, sepsis, congenital heart disease, prematurity, and intestinal malabsorption^1^.

Laparoscopic cholecystectomy (LC) has gradually become the primary technique for treating symptomatic gallstones in children^5^. Compared with open cholecystectomy, LC has several advantages, including less postoperative pain, an earlier recovery period, a quicker return to unrestricted activities, and better cosmetic outcomes^3^^,^ 9. LC is considered a safe procedure with a low rate of complications. In adults, injury to the bile duct occurs in 0.85% of cases, but in children and adolescents no index has been defined^13^^,^ 14. The most frequent causes of bile duct injury are inadequate exposure to the surgical field and failure to identify the hepatic hilum structures. Furthermore, acute or chronic gallbladder inflammation, obesity, anatomical variations, and bleeding also predispose patients to accidental injuries of the biliary tree during the surgical procedure, both in children and adults^13^^,^ 14.

Cholecystectomy is a surgical procedure not often performed in pediatric patients. Pediatric surgeons face difficulty gaining experience in this operation, which can have serious complications^15^^-^ 19.

In this study, we retrospectively reviewed the clinical and sonographic data, histopathological findings, and the outcomes in patients with symptomatic cholecystic disease who underwent LCs.

## Methods

The present study was conducted in accordance with the ethical principles of the Declaration of Helsinki and its amendments, and it was approved on December 22, 2020, by the institution’s Research Ethics Committee (Comitê de Ética e Pesquisa em Seres Humanos of the Centro Universitário of the Faculdade de Medicina do ABC, number 4.482.005).

This study is retrospective, cross-sectional, and descriptive, involving the medical records of 50 patients of both sexes aged younger than 18 years old, with symptomatic cholelithiasis, who underwent LC at the Pediatric Surgery Unit of Hospital Estadual Mário Covas (Santo André, SP, Brazil), from August 2008 to August 2019.

The primary outcome aimed to investigate the clinical characteristics and treatment of children and adolescents with symptomatic cholecystolithiasis undergoing LC at a single institution. The second outcome was to study the results of LC in children and adolescents with symptomatic cholecystolithiasis.

Routine demographic and clinical data were collected for all patients, including documented comorbidities. The clinical diagnoses of the cases included in this study were determined and classified into nonacute and acute conditions. Nonacute pathologies include biliary dyskinesia, symptomatic cholelithiasis, and others. Acute pathologies include cholecystitis and gallstone pancreatitis/choledocholithiasis. There were no cases of asymptomatic cholelithiasis. The cases of patients operated on for any other condition, such as polyps, abdominal trauma, transplantation, incidental cholecystectomy, or open cholecystectomy, or those aged 18 years old or older were not included in this series. A single surgeon performed all cases. Indication for intraoperative cholangiogram during this period was based on findings at an immediate preoperative ultrasound scanning (biliary dilatation or choledocholithiasis) or if a biliary duct anomaly was suspected but unable to be defined by magnetic resonance cholangiopancreatography (MRCP).

The indication for intraoperative cholangiogram during this period was based on findings at an immediate preoperative ultrasound scanning (the presence of biliary dilatation or the presence of choledocholithiasis) or if a biliary duct anomaly was suspected but unable to be defined by MRCP. The LC surgical procedure was performed with the standard four-port surgical approach with trans-umbilical open access, three trocars (two operative and one accessory), the cystic artery, and ligation of the duct with non-absorbable polymer clips (knots or Hem-o-lok clip), and the gallbladder removal through the umbilical access. A single surgeon performed all cases.

The variables studied were sex, age, BMI, hematological disease, and gallstone type (cholesterol or bile pigment). The BMI × age curve (z score) from the World Health Organization was adopted to determine whether a patient was obese or not. Patients with a z score greater than 3 were considered obese. The type of gallstone was identified in the anatomopathological examination report of the surgical specimen.

Considering the nature of the samples, quantitative variables were represented by absolute (*n*) and relative (%) frequency, median, and arithmetic mean with standard deviation.

## Results

Among the 50 patients evaluated in the study, the mean age was 11.4 (± 3.6) (range = 3 to 17), with a median age of 12 years old (Table 1).

**Table 1:** Characteristics of children and adolescents with symptomatic cholelithiasis undergoing laparoscopic cholecystectomy.

Variables	n	%
**Sex**		
Female	33	66
Male	17	34
**Age (years old)**		
Under 7	9	18
8 to 12	22	44
13 to 15	11	22
16 to 18	8	16
**Body mass index (median = 25)**		
Not obese (median = 18)	37	74
Obese (median = 32)	13	26
**Hematological disease**		
Absent	38	76
Present	12	24
**Type of gallstone**		
Cholesterol	25	50
Bile pigment	3	6
Not informed	22	44

Source: Elaborated by the authors.

Among patients with hematological disease, eight (16%) had sickle cell anemia, and four (8%) had hereditary spherocytosis. In the remaining 38 patients (76%), a specific cause of cholecystolithiasis could not be identified (Table 1). No comorbidities, except hematological diseases, were observed in the patients in this series.

No patient underwent clinical treatment for stone dissolution or had undergone any previous surgery. Among obese patients, nine (18%) were females, and four (8%) were males. Two obese patients (4%) had a hematologic disease. Familial hyperlipidemia or glucose-6-phosphate dehydrogenase deficiency was not observed.

In patients with a clinical picture of abdominal pain, symptoms occurred from one week to 24 months. Four patients (8%) had episodes of cholestatic jaundice: two due to acute cholecystitis, one due to choledocholithiasis, and one due to biliary pancreatitis, all without the hematological disease.

All patients underwent ultrasonography before LC, and one (2%) underwent MRCP. Preoperatively, six patients (12%) underwent endoscopic retrograde cholangiopancreatography (ERCP) with sphincterotomy (four for acute biliary pancreatitis and two for choledocholithiasis).

Thirty-eight patients (76%) underwent surgery for biliary colic or uncharacteristic abdominal pain: six (12%) for acute biliary pancreatitis, four (8%) for acute cholecystitis, and two (4%) for choledocholithiasis. All patients with acute cholecystitis underwent surgery eight weeks after the onset of symptoms. Only patients with acute biliary pancreatitis were operated on urgently.

All patients underwent LC without intraoperative cholangiography. Three cases (6%) resulted in conversion to open access due to excessive bleeding: two of them had acute cholecystitis with an intense inflammatory area between the gallbladder and the hepatic hilum, and the gallbladder firmly adhered to the liver and duodenum in the third case. In one case, an accidental lesion of the common bile duct was identified intraoperatively. This patient underwent end-to-end common bile duct anastomosis with drainage of the main bile duct with T-tube drainage. Postoperatively, the patient evolved into stenosis of the main bile duct. He underwent an ERCP, and a papillotomy with the placement of a self-expandable metallic biliary stent was performed. After 12 months, the patient was anicteric and without the biliary endoprosthesis. One patient (2%) was reoperated on for intestinal obstruction due to adhesions. Another patient (2%) had acute biliary pancreatitis and underwent an ERCP with sphincterotomy and the placement of a plastic biliary endoprosthesis. The mean length of stay was three days (range: one to 50 days).

The anatomopathological description of the gallbladder revealed the presence of chronic cholecystitis in all cases. In two cases (4%) with an initial clinical picture of acute cholecystitis, we observed the histopathological picture of an acute outbreak of chronic cholecystitis (Table 1).

There were no deaths. All patients underwent operations experience improvement or resolution of their biliary symptoms after the laparoscopic cholecystectomy.

## Discussion

In this series, the pediatric patients with cholecystolithiasis and without hemolytic disease represented most cases compared to the patients with hemolytic diseases. This tendency was also observed by other authors^3^^,^ 4 ^,^ 6 ^,^ 11 ^,^ 13 ^,^ 20 ^–^ 22. The hemolytic disease seems to make cholecystolithiasis manifest at an earlier age, unrelated to obesity, and more frequently in males^23^^–^ 26. Interestingly, even patients with hematological disease can present cholesterol stones, which shows that factors may overlap in the formation of these stones in hemolytic anemias^25^^,^ 26. Cholecystolithiasis was diagnosed in the majority in the age group of 8 to 12 years old, followed by the age group of 13 to 15, as observed in other series of cholecystectomy in children and adolescents^3^^,^ 4 ^,^ 6 ^,^ 11 ^,^ 13 ^,^ 20 ^,^ 22 ^,^ 27. In the present study, females had a significantly higher cholesterol stones than males.

The time elapsed between the onset of symptoms and the diagnosis is likely longer in children than in adults, probably due to the lower prevalence of the disease in this age group and consequent lower clinical suspicion^4^^,^ 5 ^,^ 7. Biliary colic attacks are irregular, and the intensity can vary from one episode to another. In pediatric patients, vomiting is a common symptom in up to 60% of symptomatic cases^8^^,^ 12. Abdominal pain, characteristic or not, has a higher incidence with advancing age. It can be expected that the increased prevalence of cholecystolithiasis risk factors in pediatric patients will improve the indication of cholecystectomy for conditions typically found in adult patients^7^. Abdominal ultrasonography is the method of choice for diagnosing cholecystolithiasis in adults and children^4^^,^ 5.

LC is the criterion standard for treating cholecystolithiasis in children and adolescents^9^^,^ 14 ^,^ 16 ^,^ 18 ^,^ 24 ^,^ 28. It is associated with less postoperative pain, shorter hospital stays, a quicker return to unrestricted activities, lower risk of infection, and a less intrusive scar when compared to conventional surgery. As a rule, LC is considered a safe procedure with a low rate of complications^3^^,^ 9 ^,^ 14 ^,^ 16 ^,^ 24 ^,^ 25 ^,^ 29.

Managing incidentally diagnosed and asymptomatic gallstones in the pediatric age group poses a dilemma^10^^,^ 30 ^,^ 31. Long-term follow-up studies have consistently shown that only a minority of asymptomatic gallstones lead to the development of symptoms or complications which makes performing LC challenging^5^^,^ 6. However, surgical intervention is appropriate with the presence of a clinical picture compatible with gallstone disease^2^^,^ 5 ^,^ 6 ^,^ 19 ^,^ 22. In our opinion, the disadvantage of expectant treatment is that complications may develop, which leads to more morbidity, whereas routine cholecystectomy can avoid those complications.

In the present series, complications of cholecystolithiasis were observed, such as acute cholecystitis, biliary pancreatitis, and choledocholithiasis, indicating that this condition in the pediatric population can progress to severe illnesses^8^^,^ 13 ^,^ 30 ^,^ 32. During an observation period of 17 years, Tannuri et al.^11^ verified that 25% of patients had complications from cholecystolithiasis. In the present series, in 11 years, 12 patients (24%) had complications from biliary disease: biliary pancreatitis, acute cholecystitis, and choledocholithiasis. These results emphasize the need to avoid temporizing cholecystolithiasis in that age group.

An LC with a preoperative or postoperative ERCP is at least a two-procedure process. In contrast, a cholecystectomy with a laparoscopic common bile duct exploration (LCBDE) can provide definitive treatment in a single procedure under one anesthetic^33^^,^ 34. Exploration of the common bile duct and removal of stones by an LCBDE is safe and feasible in pediatric patients to treat choledocholithiasis. Through this procedure, choledocholithiasis and cholelithiasis can be treated in a single procedure without papillotomy or fluoroscopy. Compared with an LC and ERCP, an LCBDE is associated with a shorter hospital stay^34^^–^ 36. Unfortunately, during the period in which the cases in this series were operated on, the Pediatric Surgery Unit did not have an LCBDE available.

Major postoperative complications in an LC are uncommon^4^^,^ 13 ^,^ 29, and in this series they occurred in two patients: one patient had postoperative biliary pancreatitis, and the other one patient had a bile duct stenosis due to an intraoperative injury In both cases, the patients had a good recovery and no intercurrences.

Mattson et al.^28^ performed a systematic review with meta-analysis through 114 studies and 76.524 patients. The authors found a rate of 3.4% for postoperative complications in children, slightly lower than that of the present series (4%). In their meta-analysis, the conversion rate to conventional surgery was 2 vs. 6% in our series. Despite the experience of the single surgeon, it is possible to speculate that, of the cases that were operated on urgently, they contributed to increasing the number of cases with complications or even the need to convert the LC to open access.

The main limitations of this study were its retrospective character and single-center design. Furthermore, due to the number of patients included, additional studies with a larger sample are needed to confirm the results obtained in the current series.

## Conclusion

The natural evolution of cholecystolithiasis in the pediatric population can present serious complications, emphasizing the need to avoid temporizing cholecystolithiasis in children and adolescents because LC in this age group is a safe operation with low complication rates. When necessary, a cholecystectomy with a LCBDE can provide definitive treatment in a single procedure under one anesthetic, avoiding papillotomy or fluoroscopy.

## Data Availability

All data were generated or analyzed in the current study.
